# The nature of organic records in impact excavated rocks on Mars

**DOI:** 10.1038/srep30947

**Published:** 2016-08-05

**Authors:** W. Montgomery, G. D. Bromiley, M. A. Sephton

**Affiliations:** 1 Impacts and Astromaterials Research Centre, Department of Earth Science and Engineering, Imperial College London, SW7 2AZ, UK; 2 School of GeoSciences, University of Edinburgh, Grant Institute, West Main Road, Edinburgh EH9 3JW, UK

## Abstract

Impact ejected rocks are targets for life detection missions to Mars. The Martian subsurface is more favourable to organic preservation than the surface owing to an attenuation of radiation and physical separation from oxidising materials with increasing depth. Impact events bring materials to the surface where they may be accessed without complicated drilling procedures. On Earth, different assemblages of organic matter types are derived from varying depositional environments. Here we assess whether these different types of organic materials can survive impact events without corruption. We subjected four terrestrial organic matter types to elevated pressures and temperatures in piston-cylinder experiments followed by chemical characterisation using whole-rock pyrolysis-gas chromatography-mass spectrometry. Our data reveal that long chain hydrocarbon-dominated organic matter (types I and II; mainly microbial or algal) are unresistant to pressure whereas aromatic hydrocarbon-dominated organic matter types (types III and IV; mainly land plant, metamorphosed or degraded, displaying some superficial chemical similarities to abiotic meteoritic organic matter) are relatively resistant. This suggests that the impact excavated record of potential biology on Mars will be unavoidably biased, with microbial organic matter underrepresented while metamorphosed, degraded or abiotic meteoritic organic matter types will be selectively preserved.

Indigenous, unaltered organic matter has not been conclusively recognised in Martian surface materials despite a number of in situ mission attempts at its detection^1^,^2^,^3^. This failure to observe organic matter is surprising considering the regular infall of organic rich meteoritic material onto the surface^4^. The lack of organic matter at the surface of Mars and the possible inability to detect organic matter on Mars missions has been attributed to the presence of oxidants in the Martian regolith^5^,^6^, the mineral assisted oxidation and chlorination of organic matter during thermal extraction^7^ and degradation of organic matter by cosmic rays^8^. Recent work has detected chlorinated organic material on Mars^9^, but information about the nature of the indigenous Martian organic matter prior to chlorination remains scarce.

With surface reactions proposed as sources of oxidising chemicals on Mars, the importance of sampling depth for effective life detection is recognised^10^. Oxidising materials on the Martian surface include perchlorates^11^ which cause the oxidation and chlorination of organic matter during thermal extraction^7^,^9^ and Martian sulfates have the potential to cause similar problems^12^. Sample collection which penetrates to depths that are beyond the influence of radiation^8^ and oxidation^13^ should substantially increase the probability of successful organic detection^14^. Drilling is part of forthcoming mission activities to avoid shallow depths in which organic records are likely to have been degraded. The ExoMars 2018 rover for example will drill to a maximum of 2 meters depth to obtain samples for analysis by an onboard analytical laboratory^15^.

Yet drilling is not the only way to access the Martian subsurface. Natural excavation of the Martian crust by recent impact events has been proposed as a mechanism for obtaining subsurface materials, with depth of sampling correlating with crater size and complexity; small simple craters can access depths of hundreds of meters while large complex craters can sample kilometre depths^16^,^17^. Cratering produces ejecta blankets that include both subsurface rock debris and ice, with the most recent craters providing access to host matrices and therefore any internal organic constituents that would be unstable at the surface over long timescales. Sampling impact ejecta has been proposed as a mechanism of accessing the sub surface geological diversity of Martian stratigraphy, including the presence of any biological signatures^18^. The proposed study of impact ejecta requires an understanding of any sampling bias that has been introduced by the temperatures and pressures associated with ejection. Recent ejecta would be preferable to ancient ejecta to avoid surface degradation of any exhumed biosignatures but the ejection process itself is a distinct and unavoidable preceding step that must be understood.

If detected, organic matter from once-living organisms is unlikely to represent a complete high fidelity record. Most organic records represent portions of life (e.g. selected diagnostic remnants) that can act as proxies for the complete organisms. Hence, recognising how any selective preservation mechanisms operate is essential for accurate interpretations. The most common example of selective preservation is the persistence of hydrocarbon-based organic materials relative to other structures such as nucleic acids, proteins and carbohydrates during diagenesis in terrestrial materials, and similar processes can be expected to occur on Mars.

Although the process of converting biomolecules to geomolecules (organic structures which are stable under geologic conditions) under a range of thermal conditions and timescales is relatively well understood, the possibility of selective destruction of organic structures during impact excavation is less well explored. Cratering events that transport materials from subsurface to surface are associated with great energies and organic structures are known to respond to such influences^19^,^20^. Impact energy is dispersed as heat, pressure, sound and light. Heat is a well-known modifier of organic materials and effects include the selective degradation of labile species and transformation of more resistant organic structures^21^. It has been established that high pressures can lead to changes in individual organic molecules^22^. A small number of laboratory investigations have used piston-cylinder apparatus to study the response of individual organic standards to high pressures^23^,^24^,^25^, but similar experiments at these pressures on complex natural organic assemblages are unprecedented. If impact ejected rocks are to be used as targets for biosignature detection on Mars then the influence of high pressure on organic matter must be understood. If left unaddressed, any selective effects of impact excavation would cause either an incomplete interpretation of past ecosystems or incorrect indications of the complete absence of past life.

A number of studies have shown that organic matter can be preserved following impact events. Most common are studies intended to investigate the possibility of panspermia, the transfer of life between planetary bodies, which also provide a guide to whether this life would survive planetary bombardment^26^,^27^,^28^,^29^. These studies commonly subject living material (i.e., bacteria) to impact shocks and make some measure of survivability demonstrated by maintenance of the ability to reproduce. A recent survey of work in this area gives survival rates ranging from between 0.000008 and 0.9%^30^. Although these studies provide useful insight into bacterial survivability they do not offer information on which biosignatures will be encountered during future planetary missions that sample and analyse exhumed fossil material.

Another approach commonly taken is to investigate specific organic compounds under impact-generated pressures in the laboratory. Compounds are typically chosen because they either have been observed in the relevant extraterrestrial environments (e.g. carbon, water, ammonia and nitrogen as in Furukawa *et al*.^31^) or as suitable proxies in the absence of direct chemical observations (e.g. anthracene and stearic acid as in Burchell *et al*.^20^). Such studies of simple molecular systems and mixtures typically focus on synthesis of larger molecules from these smaller compounds, or the survivability of individual molecules.

Fossil biomarkers are a class of organic molecules of specific interest to life detection. Impact studies have examined fossil biomarkers and they have been recognised within rocks from the Haughton impact melt breccia and carbonate bedrock^32^, and are also preserved in shale within impact breccias at the Ries impact crater^33^. Laboratory studies have demonstrated that following hypervelocity impact of organic-rich rocks, fossil organic biomarkers are not heated to above carbon-carbon bond breaking temperatures and so their information-rich hydrocarbon backbones can survive impact events^19^. Although pressure effects are heterogeneously distributed in the subsurface during and following impacts^17^, data on the general relative survivability of different types of organic matter and particularly organic matter present as natural mixtures under a range of pressures represent valuable information.

The chemical structure of organic matter in the geosphere reflects its ultimate biological source. The long chain hydrocarbon-dominated units of type I and II organic matter reflect microbial or algal inputs. The aromatic ring and short chain hydrocarbon-dominated type III and IV organic matter assemblages reflect land plant biopolymers or the organic residues of degradation (Supplementary Information). Owing to their microbial inputs, type I and type II organic materials can help predict the responses of organic chemical classes that may be expected from microbial remains of primitive Martian life. By contrast, organic materials from evolutionarily advanced land plants are unlikely on Mars but their responses to impact processes are still useful in the search for organic signals of life on the red planet. Type III and IV organic materials share some chemical structures with the dominant macromolecular component of meteorite organic matter and have a history of use as analogues for chemical investigations of meteorites^34^,^35^. Hence, the responses of type III and IV organic materials to impact processes can also suggest the fate of important abiotic organic inputs from meteorite infall. Moreover type IV organic matter in particular may reflect the nature of some organic fossils when exposed to the oxidant and radiation-rich near surface of Mars.

Owing to their importance as the source of petroleum and coal, types I, II and III organic matter have been studied extensively under varying temperature conditions and depositional environments. The kinetics of these systems have been extensively explored and scaling between laboratory and natural environments is generally well understood. The stability of these types of organic matter under the pressure and temperature conditions of impacts, large enough to unearth material for sampling, however, remains largely unknown.

To test the preservation of various types of organic matter, we subjected organic-rich sedimentary samples to temperature and pressure conditions comparable to an impact. We obtained pyrolysis gas chromatography-mass spectrometry (Py-GC-MS) data for both untreated and treated organic matter. The molecular products of pyrolysis access both the soluble and insoluble organic matter contained within the whole rock. We compared the pre- and post- pressurization state of the various organic matter types to reveal how each type responds to the pressures associated with impact excavation. It was our aim to document those classes of structures which selectively respond to pressure and which are therefore most likely to be involved in sampling bias in impact-ejected rocks. Hence, our focus is on the obvious survival and destruction preferences of general compound classes.

## Results and Discussion

**Figure 1:**
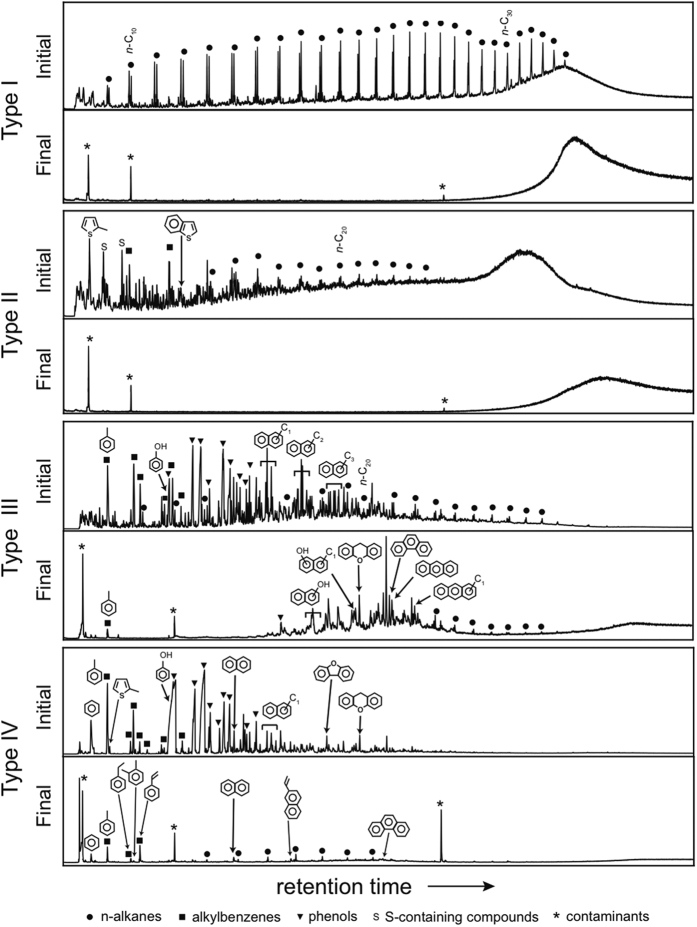
Total ion current chromatograms of pyrolysis products of type I, II, III, and IV organic materials before (initial) and after (final) pressure treatment. Type I and II organic materials are rich in straight chain hydrocarbons and are destroyed by pressure treatment. Type III and IV organic materials are rich in cross linked aromatic units and are relatively resistant to pressure. Type III and IV organic materials or are more likely to survive impact ejection from the subsurface.

**Figure 2:**
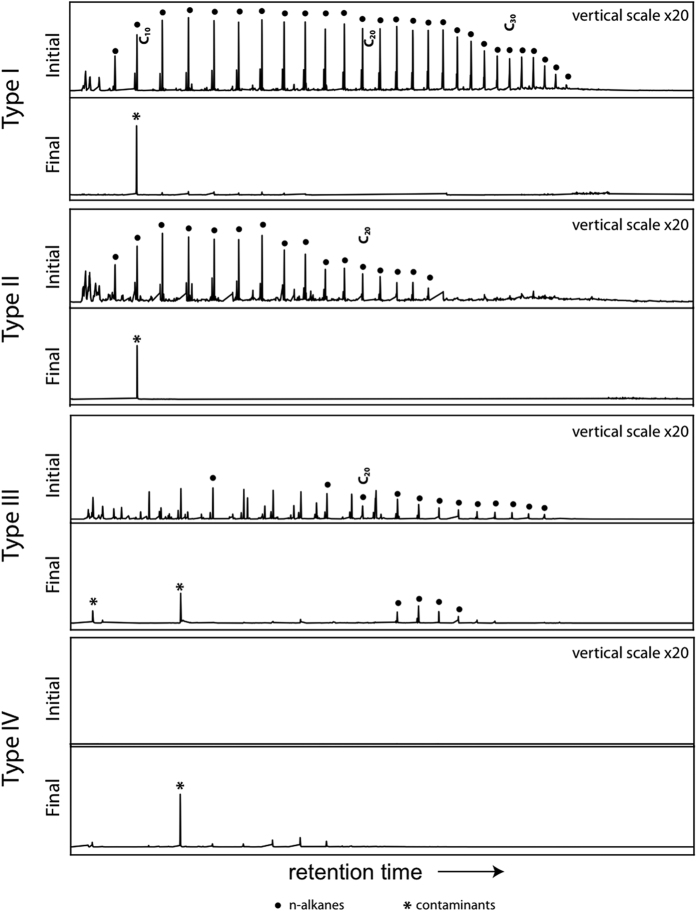
Extracted ion chromatograms (m/z 57) of pyrolysis products of type I, II, III, and IV organic materials before (initial) and after (final) pressure treatment. The m/z 57 ion selectively highlights the presence of aliphatic hydrocarbons and displays the susceptibility of straight chain hydrocarbons to destruction by pressure (present in the initial samples but absent in the final pressure treated samples). The series of peaks in the starting materials represent *n*-alkene/*n*-alkane doublets, which are the pyrolysis products of high molecular weight aliphatic networks. All vertical scales have been expanded x20 relative to Fig. 1.

Following online pyrolysis, types I and II organic matter produce numerous alkene/alkane doublets that reflect highly aliphatic biopolymers (Fig. 1). The presence of aliphatic pyrolysis products can be highlighted by the use of extracted ion chromatograms in which the m/z = 57 ion is characteristic of alkanes (Fig. 2). Identifications of the main structures in the starting material are presented in Fig. 1 and listed in Table 1. Pyrolysis of the type I and II organic matter following pressure-temperature treatment produces only a few products: toluene, decane and phthalates, which are most likely laboratory or storage contaminants. This is in direct contrast to anhydrous confined pyrolysis experiments performed on Type I organic matter at 450 °C/16 hours, which showed the production of alkanes, aromatic and polar compounds^36^, a result consistent with similar 72 hour experiments run on Type I organic matter at 400 and 500 °C^37^. Anhydrous confined pyrolysis experiments on Type II organic matter showed the promotion of aromatization (e.g., the formation of naphthalenes, methylated naphthalenes, etc.) at this temperature and low pressures (est. 4–140 MPa)^38^,^39^,^40^. The data suggest that the original aliphatic materials are no longer amenable to visualization by online pyrolysis following the application of pressure and their organic signals are, therefore, unlikely to be accessible following impact ejection.

The online pyrolysis of types III and IV organic materials liberates numerous aromatic and phenolic units (Fig. 1, detailed assignments given in Table 1). Aromatic and polar polymers appear more likely than aliphatic hydrocarbons to remain accessible by online pyrolysis when subjected to pressure and should survive impact ejection. Owing to the presence of Type III organic matter in coal there are many studies reporting the results of anhydrous pyrolysis at 450 °C (and higher) and a range of organic compounds including alkanes, aromatic hydrocarbons and polar compounds has been observed for a variety of durations and heating rates^41^. Notably, our pressure-treated Type III sample lacks a low molecular weight fraction when compared to similar samples which have been artificially matured with temperature alone, suggesting that these organic structures have either volatilized or formed additional cross linkages which render them unresponsive to online pyrolysis analysis. Type IV kerogens are characterised by their relative inability to produce organic products in response to thermal processing including online pyrolysis.

The relative survivability of aromatic and polar molecules is consistent with reports of fossil biomarkers in terrestrial impacts such as the Haughton and the Ries craters^32^,^33^. Although the observations were made in the crater walls instead of the impact ejecta, the biomarkers in the Haughton and Ries rocks, which contain some aromatic, have survived the considerable shock pressures associated with these impact events.

Our results are in harmony with other published works that indicate the increased resistance of aromatic units to temperature and pressure: aromatic hydrocarbons in the form of polycyclic aromatic hydrocarbons have been extensively studied and found to be stable to pressures and temperatures in excess of those encountered in our study^22^. A few investigations have been carried out using static and shock pressure techniques on phenol and similar compounds, which show this class of molecules to be stable at high pressure^42^, but a mechanism for this stability has not yet been proposed. Aliphatic polymers have lower densities, elastic moduli and tensile strength relative to their more mechanically stable highly cross linked aromatic counterparts^43^, which may explain their relative instability at high pressure.

Our work shows organic assemblages in impact-ejected rocks on Mars could suffer from two stages of preservation bias. During initial burial the more easily degradable components such as nucleic acids, proteins and carbohydrates and their monomers would be progressively lost, leaving a hydrocarbon-rich residue that may contain aliphatic and aromatic units. During impact ejection from the subsurface a second preservation bias could selectively concentrate aromatic and polar molecules relative to their aliphatic counterparts, which are either volatilized or transformed into analytically unamenable macromolecular material. These effects of pressures associated with impact ejection will make detection of organic remnants of microbes more difficult unless their organic matter has been aromatised under the influence of post burial maturation. In the context of Mars, where both biotic and abiotic organic inputs may be relevant, the consequences of such a preservation bias in impact ejected rocks may be to selectively preserve degraded and unrecognizable fossil organic matter or highly aromatic meteorite macromolecules. Examination, therefore, may superficially and incorrectly imply a world dominated by non-biological organic chemistry from meteorite sources.

The relationship between the high pressures experienced by impact ejecta and our predicted observation of dominantly aromatic organic matter can be tested by reference to studies of the organic matter in Martian meteorites. All Martian meteorites are impact-ejected fragments of the subsurface. Online pyrolysis of the curated Martian meteorites would be expected to produce aromatic units. Published organic geochemical studies indicate that there is organic matter present in Martian meteorites and that it is dominantly aromatic in nature^44^,^45^. No abundant aliphatic material has been detected in Martian meteorites suggesting its absence prior to, or destruction during, the impact events which propelled these rocks towards Earth. While there is a report of aliphatic amino acids detected in a Martian meteorite^46^, the authors conclude the likely sources are secondary processes.

The Martian subsurface is more likely to have preserved organic matter than the surface owing to the latter environment being subjected to radiation and oxidation. Impact excavation provides access to the subsurface without costly and difficult drilling. However, the pressure associated with impacts can selectively hide particular types of organic structure from detection using online pyrolysis, with aliphatic hydrocarbons, the most likely indicators of primitive biological organic chemistry, most strongly affected. Impact events which excavate organic records from depth will, therefore, produce a sampling bias: microbial organic matter may be rendered undetectable by impact-associated pressure, while thermally metamorphosed, degraded or meteoritic organic matter could be preferentially preserved. The effects of pressure on the fidelity of organic records of potential past Martian biology must be appreciated during future life detection missions to the red planet.

## Materials and Methods

### Samples

Four organic matter types were selected for the pressure experiments (details given in Table 2 and Appendix A). Type I organic matter was represented by a black shale of Lower Carboniferous age collected from Port Edgar, west Lothian, Scotland, UK^47^. Type II organic matter was represented by a black shale sample of the Blue Lias of Lower Jurassic age collected from Monmouth Beach, Dorset, UK^48^. Type III organic matter was represented by a high volatile bituminous coal from Saarland, Germany^49^. Type IV organic matter was a sample of charcoal obtained from the Upper Greensand Formation at Durdle Door, Dorset, UK^35^. The level of thermal metamorphism (maturity) experienced by the samples can be indicated by vitrinite reflectance values (VRo%), a maturity indicator based on reflected light from land plant fragments (vitrinite), and range from the start (0.6) to the middle (0.9) of the “oil window,” the maturity range in which oil is generated from organic-rich source rocks. Although provided for completeness, maturity is not expected to have an influence on interpretation of the experiments given that they are designed to examine differences in organic matter both before and after the application of pressure. The whole rock samples were ground to homogenize them and to facilitate loading into the piston-cylinder apparatus and pyrolysis tubes.

### High pressure piston-cylinder experiments

Small quantities of each whole rock samples containing representatives of the various organic matter types were crushed and ground into fine powders in an agate pestle and mortar, then packed into 5 mm o.d., 0.3 mm wall thickness, 5 mm long gold capsules, which were then welded shut. Capsules were cooled with liquid N_2_ during welding to prevent unwanted heating and degradation of the samples. Capsules were then placed into talc-pyrex sample assemblies with internal graphite resistance furnaces^50^ and subsequently run in an end-loaded type piston-cylinder apparatus at 0.5 GPa, 450 °C. As these conditions are at the low pressure/temperature working range of the piston-cylinder apparatus, experiments were run for 3 days, according to normal lab protocol to ensure accurate pressure calibration within the range +/−0.1 kbars; however, under these conditions thermal equilibrium of samples under run conditions is expected within a timescale of minutes^51^. All experiments were run using the hot-piston out technique^52^. Pressure was calibrated to within ±0.1 kbar using the quartz-coesite transition and albite = jadeite + quartz reaction. Temperature was measured throughout experiments using an R-type thermocouple placed adjacent to the capsule within the assembly. Experiments were quenched to room temperature in less than 10 seconds by turning off power to the heating circuit. Recovered capsules were then carefully sliced and samples removed.

Experimental conditions used in this study were chosen to assess effects of smaller, recent, <10 meter-sized impacts sufficient to excavate and make accessible regions of the Martian subsurface where organic material is hypothesised to be present (a depth of >2 m)^14^,^53^. On the basis of systematic assessment of the response of terrestrial materials to varying shock conditions (French^54^; their Table 4.2 and subsequent discussion), we make the specific assumption that materials respond during impact events by attaining thermodynamic equilibrium at close to peak shock conditions. The results of complex numerical simulations suggest that our experimental conditions may reflect the pressures experienced by the crust on reaching peak temperature (not peak shock pressure)^55^,^56^. The post shock thermal regime is expected to be <100 °C^54^, insufficient to alter any organic material which has survived the higher temperatures associated with the main event.

### Pyrolysis-gas chromatography-mass spectrometry (Py-GC-MS)

Py-GC-MS is a common technique used for organic detection on Mars^1^. While there are differences in heating rate (20 °C ms^−1^ vs 35 °C m^−1^, respectively) and environmental conditions (~1013 and ~25 mbar He respectively) between laboratory- and rover- based pyrolysis instrumentation, the techniques are the same and have similar effects on organic material; the difference in heating rates could affect the distribution of pyrolysis products but this does not change our interpretation^7^. For online pyrolysis, whole rock samples were placed in quartz sample tubes and loaded into a CDS Analytical Model 5200 pyrolysis, then subjected to flash heating at 650 °C at a heating rate of 20 °C ms^−1^. The pyrolysis products were introduced to an Agilent 6890 gas chromatograph using split injection at a 50:1 split ratio and an inlet temperature of 250 °C. Separation was performed on a 30 m J&W Scientific DB-5MS Ultra Inert column. The oven temperature program comprised a start temperature of 50 °C held for 1 minute, followed by a ramp of 4 °C min^−1^ to 310 °C where the temperature was held for 20 min. Helium column flow was 1.1 ml min^−1^. Post-separation compound identification took place using an Agilent 5973 inert Mass Selective Detector, which collected data over a scan range of m/z = 50 to 550. Assignments are made by considering the elution order against published work and comparing mass spectra against a standard library (NIST08).

## Additional Information

**How to cite this article**: Montgomery, W. *et al*. The nature of organic records in impact excavated rocks on Mars. *Sci. Rep.* **6**, 30947; doi: 10.1038/srep30947 (2016).

## Supplementary Material

Supplementary Information

## Figures and Tables

**Table 1:** Compound identifications in pyrolysis products of types I to IV organic materials both before and after high pressure treatment.

Sample	Before pressure treatment	After pressure treatment
Type I	C_9_-C_35_ alkenes/alkanes	toluene (contaminant)
	C_9_-C_34_ alkenes/alkenes	decane (contaminant)
		pthalic acid ester (contaminant)
Type II	C_9_-C_25_ alkenes/alkanes	toluene (contaminant)
	C_1_-thiophenes	decane (contaminant)
	C_2_-thiophenes	pthalic acid ester (contaminant)
	C_3_-thiophenes	
	C_4_-thiophenes	
	C_5_-thiophenes	
	C_3_-benzenes	
	C_4_-benzenes	
	benzothiophene	
Type III	C_5_-C_30_ alkenes/alkanes	dichloromethane (contaminant)
	C_9_-C_34_ alkenes/alkenes	decane (contaminant)
	toluene	pthalic acid ester (contaminant)
	phenol	C_22_-C_26_ alkenes/alkanes
	C_2_-benzenes	toluene
	C_3_-benzenes	naphthol
	C_1_-phenols	C_1_-naphthols
	C_2_-phenols	xanthene
	naphthalene	anthracene
	C_1_-naphthalene	phenanthrene
	C_2_-naphthalene	C_1_-anthracene
	C_3_-naphathalene	
Type IV	benzene	dichloromethane (contaminant)
	toluene	decane (contaminant)
	C_1_-thiophene	pthalic acid ester (contaminant)
	C_2_-benzenes	benzene
	C_3_ benzene	toluene
	phenol	C_2_-benzenes
	methoxy-methyl benzene	naphthalene
	C_1_-phenols	C_2_-naphthalenes
	benzofuran	phenanthrene
	C_2_-phenols	
	naphthalene	
	C_4_-phenols	
	C_1_-naphthalenes	
	dibenzofuran	
	xanthene	

Note that background contaminants become more prominent when the indigenous material has been removed.

**Table 2:** Details of samples used for the high pressure experiments.

Sample		Location	Age	TOC (%)	VRo%	Ref.
Type I	Lacustrine shale	Port Edgar, west Lothian, Scotland, UK	Carboniferous	13.43	0.9	47
Type II	Marine shale	Monmouth Beach, Dorset, UK	Jurassic	8.14	0.6	48
Type III	High volatile bituminous coal	Schwalbach Coal Seam, Ensdorf Colliery, Saarland, Germany	Carboniferous	56.4 (C%)	0.79	49
Type IV	Charcoal	Wealden Beds, Durdle Door, Dorset, UK	Cretaceous	100	—	35
