# Supplementary material for: The nature of organic records in impact excavated rocks on Mars
Source: Sci Rep. 2016 Aug 5;6:30947. doi: 10.1038/srep30947 (PMC4974657; doi:10.1038/srep30947)
Supplement: Supplementary Information [file srep30947-s1.pdf]

# The nature of organic records in impact excavated rocks on Mars

W. Montgomery*[1], G. D. Bromiley[2], and M. A. Sephton[1]

[1]Impacts and Astromaterials Research Centre, Department of Earth Science and Engineering, Imperial College London, SW7 2AZ, UK

[2]School of GeoSciences, University of Edinburgh, Grant Institute, West Main Road, Edinburgh EH9 3JW, UK

* Corresponding author: Wren Montgomery, Department of Earth Science and Engineering, Imperial College London, South Kensington Campus, London, SW7 2AZ, UK. Tel: +44 (0)2075945185; Email: w.montgomery@imperial.ac.uk.

Figure A.1:

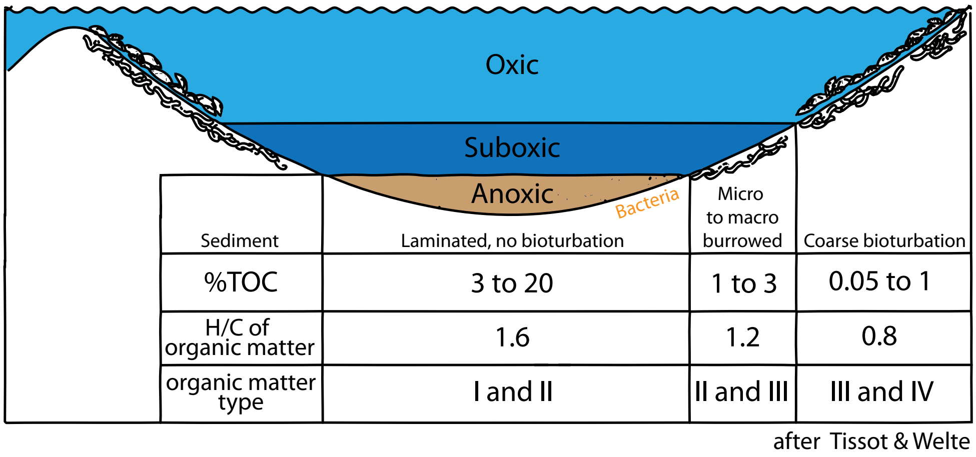


Supplementary Figure 1: Varying organic type with depositional environment, after Tissot & Welte[1].

1 Tissot, B. P. & Welte, D. H. *Petroleum Formation and Occurrence*. (Springer-Verlag, 1984).
